# Supplementary material for: Resolving the full spectrum of human genome variation using Linked-Reads
Source: Genome Res. 2019 Apr;29(4):635–45. doi: 10.1101/gr.234443.118 (PMC6442396; doi:10.1101/gr.234443.118)
Supplement: Supplemental Material [file supp_gr.234443.118_Supplemental_longranger-2.2.zip › longranger-2.2.tar.bz2!/lr2.2-public/lib/stan/cmdstan/stan/doc/api/html/model__header_8hpp.html]

Stan: src/stan/model/model\_header.hpp File Reference


|  |  |
| --- | --- |
|  | Stan  2.5.0  probability, sampling & optimization |


- Main Page
- Related Pages
- Namespaces
- Classes
- Files

- File List
- File Members

All Classes Namespaces Files Functions Variables Typedefs Enumerations Enumerator Friends Macros Pages

- src
- stan
- model

model\_header.hpp File Reference

`#include <cmath>`  
`#include <cstddef>`  
`#include <fstream>`  
`#include <iostream>`  
`#include <sstream>`  
`#include <stdexcept>`  
`#include <utility>`  
`#include <vector>`  
`#include <boost/exception/all.hpp>`  
`#include <boost/random/linear_congruential.hpp>`  
`#include <stan/agrad/rev.hpp>`  
`#include <stan/agrad/rev/matrix.hpp>`  
`#include <stan/agrad/fwd.hpp>`  
`#include <stan/agrad/fwd/matrix.hpp>`  
`#include <stan/io/cmd_line.hpp>`  
`#include <stan/io/dump.hpp>`  
`#include <stan/io/reader.hpp>`  
`#include <stan/io/writer.hpp>`  
`#include <stan/io/csv_writer.hpp>`  
`#include <stan/math/matrix.hpp>`  
`#include <stan/math.hpp>`  
`#include <stan/math/rep_array.hpp>`  
`#include <stan/math/rep_vector.hpp>`  
`#include <stan/math/rep_row_vector.hpp>`  
`#include <stan/math/rep_matrix.hpp>`  
`#include <stan/model/prob_grad.hpp>`  
`#include <stan/prob/distributions.hpp>`  
`#include <stan/common/command.hpp>`

Go to the source code of this file.


---

[ Stan Home Page ]

*© 2011–2014,
Stan Development Team.*
